# Correction for Rando et al., “Pathogenesis, Symptomatology, and Transmission of SARS-CoV-2 through Analysis of Viral Genomics and Structure”

**DOI:** 10.1128/msystems.01447-21

**Published:** 2022-01-25

**Authors:** Halie M. Rando, Adam L. MacLean, Alexandra J. Lee, Ronan Lordan, Sandipan Ray, Vikas Bansal, Ashwin N. Skelly, Elizabeth Sell, John J. Dziak, Lamonica Shinholster, Lucy D’Agostino McGowan, Marouen Ben Guebila, Nils Wellhausen, Sergey Knyazev, Simina M. Boca, Stephen Capone, Yanjun Qi, YoSon Park, David Mai, Yuchen Sun, Joel D. Boerckel, Christian Brueffer, James Brian Byrd, Jeremy P. Kamil, Jinhui Wang, Ryan Velazquez, Gregory L. Szeto, John P. Barton, Rishi Raj Goel, Serghei Mangul, Tiago Lubiana, COVID-19 Review Consortium, Anthony Gitter, Casey S. Greene

**Affiliations:** a Department of Systems Pharmacology and Translational Therapeutics, University of Pennsylvania, Philadelphia, Pennsylvania, USA; b Department of Biochemistry and Molecular Genetics, University of Colorado School of Medicine, Aurora, Colorado, USA; c Center for Health AI, University of Colorado School of Medicine, Aurora, Colorado, USA; d Department of Quantitative and Computational Biology, University of Southern California, Los Angeles, California, USA; e Institute for Translational Medicine and Therapeutics, Perelman School of Medicine, University of Pennsylvania, Philadelphia, Pennsylvania, USA; f Department of Biotechnology, Indian Institute of Technology Hyderabad, Sangareddy, Telangana, India; g Biomedical Data Science and Machine Learning Group, German Center for Neurodegenerative Diseases, Tübingen, Germany; h Perelman School of Medicine, University of Pennsylvania, Philadelphia, Pennsylvania, USA; i Institute for Immunology, Perelman School of Medicine, University of Pennsylvania, Philadelphia, Pennsylvania, USA; j Edna Bennett Pierce Prevention Research Center, The Pennsylvania State University, University Park, Pennsylvania, USA; k Mercer University, Macon, Georgia, USA; l Department of Mathematics and Statistics, Wake Forest University, Winston-Salem, North Carolina, USA; m Department of Biostatistics, Harvard School of Public Health, Boston, Massachusetts, USA; n Georgia State University, Atlanta, Georgia, USA; o Innovation Center for Biomedical Informatics, Georgetown University Medical Center, Washington, DC, USA; p St. George’s University School of Medicine, St. George’s, Grenada; q Department of Computer Science, University of Virginia, Charlottesville, Virginia, USA; r Department of Bioengineering, University of Pennsylvania, Philadelphia, Pennsylvania, USA; s Department of Orthopaedic Surgery, Perelman School of Medicine, University of Pennsylvania, Philadelphia, Pennsylvania, USA; t Department of Clinical Sciences, Lund University, Lund, Sweden; u University of Michigan School of Medicine, Ann Arbor, Michigan, USA; v Department of Microbiology and Immunology, Louisiana State University Health Sciences Center Shreveport, Shreveport, Louisiana, USA; w Azimuth1, McLean, Virginia, USA; x Allen Institute for Immunology, Seattle, Washington, USA; y Department of Physics and Astronomy, University of California-Riverside, Riverside, California, USA; z Department of Clinical Pharmacy, School of Pharmacy, University of Southern California, Los Angeles, California, USA; aa Department of Clinical and Toxicological Analyses, School of Pharmaceutical Sciences, University of São Paulo, São Paulo, Brazil; bb Department of Biostatistics and Medical Informatics, University of Wisconsin-Madison, Madison, Wisconsin, USA; cc Morgridge Institute for Research, Madison, Wisconsin, USA; dd Childhood Cancer Data Lab, Alex’s Lemonade Stand Foundation, Philadelphia, Pennsylvania, USA

## AUTHOR CORRECTION

Volume 6, no. 5, e00095-21, 2021, https://doi.org/10.1128/mSystems.00095-21. The author byline and affiliations should appear as shown in this correction.

Page 3: The following should be added to the Fig. 1 legend. ‘This figure was adapted from “Human Coronavirus Structure,” by BioRender.com (2020), retrieved from https://app.biorender.com/biorender-templates.’

Page 21: In the 2nd paragraph of Acknowledgements, “S.M.B. is currently an employee at AstraZeneca, Gaithersburg, MD, USA, and may own stock or stock options; work was initially conducted at Georgetown University Medical Center, with writing, reviewing, and editing continued while working at AstraZeneca. Y.P. is now employed by Pfizer (subsequent to contributions to this project).” should read “S.M.B. is currently an employee at AstraZeneca, Gaithersburg, MD, USA, and may own stock or stock options. Y.P. is affiliated with Pfizer Worldwide Research; the author has no financial interests to declare and contributed as an author prior to joining Pfizer, and the work was not part of a Pfizer collaboration nor was it funded by Pfizer.”

